# The 2020 Covid-19 pandemic and global value chains

**DOI:** 10.1007/s40812-020-00162-x

**Published:** 2020-06-08

**Authors:** Roger Strange

**Affiliations:** Centre for International Business and Development, University of Sussex Business School, Brighton, UK

**Keywords:** Covid-19 virus, Pandemic, Global value chains, Resilience, Location, Governance, F230

## Abstract

This paper considers the impact of the Covid-19 pandemic on firm strategies and, in particular, the configuration of firms’ global value chains (GVCs) once the pandemic has been brought under control. The merits of alternative location strategies (international diversification vs reshoring) are compared, as are the merits of different governance arrangements (internalization vs externalization) for GVC activities. The possibility of fire-sale foreign direct investment is raised, and the wider geopolitical context is emphasized. The widespread human tragedy is noted, as is the dilemma facing national governments around the world in balancing lives and livelihoods.

## Introduction

There will come a time when the 2020 Covid-19 pandemic will be (to a greater or lesser extent) under control.^1^ Many people will have died in the meantime, many others will have lost friends and/or family members, and many businesses will have ceased to exist. But for those of us fortunate to survive, lockdowns, social distancing and other such measures will be memories, and our lives will return to a degree of normality. The timescale is still very uncertain at the time of writing (mid-April 2020), but the objective of this paper is to consider the medium-term impacts on firm strategies and, in particular, the configuration of firms’ global value chains (GVCs) once all pandemic-related restrictions have been removed.

The Covid-19 pandemic has three essential features. First, it is a global phenomenon in that the virus has been detected in most countries around the world. About half of humanity (c4.5bn people) was under some form of containment in the initial stages of the pandemic.^2^ This feature differentiates the Covid-19 pandemic from other recent virus outbreaks^3^ whose health effects have been more limited and localized. As the Imperial College COVID-19 Response Team (2020: 3) comment, the ‘last time the world responded to a global emerging disease epidemic of the scale of the current Covid-19 pandemic with no access to vaccines was the 1918–19 H1N1 influenza pandemic.’ Second, the effects of the pandemic have been multi-dimensional in that it has had adverse impacts both upon public health and upon economic activity in most national economies. Furthermore, policy responses designed to address one adverse impact typically exacerbate the other: lockdowns slow the spread of the virus but harm the economy, whilst allowing people back to work benefits the economy but may lead to more infections. This differentiates the pandemic from financial crises where potential remedies were easier to conceive and match to the underlying problems. Third, the pandemic is contagious not just in the health sense but also in an economic sense, as the global economy is so inter-connected through GVCs and international movements of people, capital, goods and services. Trade in intermediate goods and services account for over 60% of total international trade Contractor (2020) reports that multinational enterprises (MNEs) were involved either as exporters or as importers, or as lead firms in GVCs, in 80 percent of all world trade (amounting to around $US 20 trillion in 2019). He further reports that the same MNE was both the exporter and the importer (i.e. simultaneously on both ends of the shipment) in approximately 40 percent of world trade. The corollary is that no country is immune to the health and economic impacts of the virus unless it is totally isolated from the rest of the world.

The structure of the paper is as follows. In the next section, we briefly consider the public health impact of the pandemic, and highlight the difficult dilemma that governments worldwide face in trying to balance the health and economic effects on their countries in the short-term. Our intention is not to address this dilemma, both because we do not have the necessary data to make informed judgments and because, ultimately, the choices will be political decisions. We then outline the short-term supply and demand shocks that the pandemic has inflicted on national economies. We next summarize the benefits for firms from participation in GVCs in normal times, and the problems that ensue when people and goods cannot move freely during pandemics. Next, we address how firms might adjust their strategies once the Covid-19 pandemic has been brought under control, particularly with regard to the configuration of the GVCs in terms of the locations of the different activities and their governance. We conclude by emphasizing the geopolitical context within which the pandemic is proceeding, and the obvious (but maybe overlooked) point that the virus needs to be brought under control everywhere not just in individual countries.

## The public health impact of the Covid-19 virus

The basic facts about the Covid-19 virus are that it is highly contagious through social contact, that those infected may show few (or even no) symptoms or may be badly (even fatally) affected, and that those infected may or may not develop immunity. The symptoms appear to be more severe for older people with pre-existing conditions, though many young and healthy people have also been affected. Extensive efforts are being made around the world to develop a vaccine. But it is likely that there will be a significant delay (months? years?) involved in designing, testing, mass producing, and administering such a vaccine.

The public health impact of the virus means that governments around the world are caught between the proverbial “rock and a hard place”: they are trying to balance lives and livelihoods. On the one hand, they are quite rightly concerned to minimize the number of infections (and the adverse health effects), to slow down the spread of the virus so that their health systems are able to cope, and to reduce the numbers of re-infections. Absent the successful deployment of an effective vaccine, this has involved teams of epidemiologists forecasting the spread of the virus. But these forecasts are inevitably sensitive to the modus operandi of the virus, the underlying assumptions regarding the social behaviour of populations, and the counter-measures introduced by governments. Different countries responded to the onset of the pandemic in different ways, at different rates, and implemented counter-measures (quarantines, lockdown, self-isolation, social distancing, closure of non-essential businesses, mass testing (both for infection and for antibodies), contact tracing, required use of facemasks and other protective equipment, travel restrictions) of varying severity. Most countries introduced draconian restrictions on travel across their national borders,^4^ and many also implemented internal restrictions. Some governments reacted quickly and implemented counter-measures whilst the number of confirmed cases were relatively small; other governments were slower.

## The effects on national economies

Perhaps the most obvious short-term effect of the counter-measures will be the supply-side impact on productive activities as employees are restricted from accessing and/or travelling to their workplaces (Wren-Lewis 2010). Some people will be able to work remotely from home, but this will not be possible in many sectors. People in the retail and hospitality sectors (restaurants, cafes, bars, hotels etc.) will be particularly hit, as will those employed in a variety of service industries (e.g. public transportation, hairdressing and beauty salons, cultural and sports events, leisure centres) where close personal contact is required. Furthermore, some people will also become infected by the virus, and will be unable to work for extended periods of time.^5^ Others may be unwilling to work for fear of becoming infected by the virus. Furthermore, school closures will mean that many parents will be unable to work as effectively either in their workplaces or even from home (Wren-Lewis 2010). There will also be indirect effects on suppliers of intermediate goods and services, as buyers cancel orders and/or extend their payment periods. These indirect effects will be larger when the buyers are large firms who can exploit the power asymmetries in their GVCs. These supply shocks are thus likely to fall disproportionately on small and medium-sized enterprises (SMEs) and their employees, and on self-employed people: these groups typically have limited cash reserves, and may be unable to weather the pandemic financially. In contrast, some sectors (e.g. e-commerce firms, delivery firms) will thrive.

On the demand side, consumption and purchasing patterns will change. These demand shocks will complement and reinforce the supply-side shocks. Firms and individuals will adjust their demand for a whole range of products. Demand for many products will fall (e.g. non-essential consumer goods, oil) even when there are no supply restrictions. Much consumer expenditure is “social” in that it involves close contact with other people (e.g. visits to restaurants and bars, attendance at sports events, concerts and theatre performances) and will be negatively affected by the pandemic (Wren-Lewis 2010). Demand for other products (e.g. streaming services) will rise. Home food consumption will increase as a result of home-working and lockdown measures, and may be exacerbated by stockpiling. Consumer shopping in town centres and retail parks will fall, but online shopping and delivery will increase. These changes in consumer behaviour will require adaptations to distribution networks, and these adaptations may take time to come into effect resulting in localized shortages.

Now governments can (and many have done) try and mitigate these shocks through combinations of macroeconomic stimulus packages (e.g. lower interest rates and fiscal stimuli), and direct support for businesses, employees and self-employed people. Perhaps inevitably, these efforts will not cover everyone and will not cover the full financial impact of the shocks. It is likely that many SMEs will go out of business unless they are provided with short-term bridging finance. Disposable income, and hence expenditure, will be lower. Share prices have already fallen, reflecting concerns about current business prospects and future uncertainties (Wagner 2020), and this too will depress consumer expenditure.

In due course, governments will try to relax their counter-measures and their economies will recover, at least in part. There will sadly be many people who will not survive the pandemic, and also businesses which will cease to trade. But schools will reopen, travel will be permitted, and most people will go back to their original working arrangements. Furthermore, consumption—and, in particular, social consumption—will resume. But many governments will have incurred substantial debts, and these will need to be funded by long-term borrowing, increased taxation and/or reduced public spending.

## The advantages and disadvantages of GVC participation

As noted above, the contemporary global economy in highly inter-connected through GVCs.^6^ At the aggregate level, this inter-connectedness is reflected in flows of foreign direct investment (FDI) and the high (60%+) proportion of global trade accounted by cross-border trade in intermediate goods and services. From a micro firm-level perspective, GVCs are characterized by various linked activities undertaken by firms in different countries. Some GVCs are characterized by numerous small firms each carrying out specific activities that are coordinated through arm’s length transactions. Other GVCs may involve many activities being internalized within large MNEs, although even highly-integrated firms will still need to buy in some inputs and may also rely on independent distributors.

There are many good reasons why firms choose to participate in GVCs in normal times. First, it is often the case (particularly in more advanced economies) that inputs of intermediate goods and services from abroad may be cheaper than similar inputs sourced from the domestic economy. These cost advantages may be due to lower labour etc. costs in foreign locations, but may also reflect differences in climate (e.g. in food production) and natural resources. Second, there may not be enough productive capacity in the domestic economy to provide the necessary inputs in sufficient quantity, or inputs of the requisite quality. Third, diversified global sourcing not only reduces firms’ unsystematic risks, but also provides them with greater resilience to supply chain disruptions. Firms which are exclusively reliant on inputs from their domestic economies are vulnerable to local disruptions such as local epidemics, strikes, hurricanes, floods, terrorist threats etc. Last but not least, consumers value the greater choice offered by the availability of final goods from foreign sources. On the debit side, GVCs bring additional costs (compared to domestic value chains) in terms of higher transportation costs, extended delivery times, and greater complexity—and these additional transaction costs will tend to rise with the different dimensions of distance (Ghemawat 2001). But the proliferation of GVCs suggests that the potential benefits of GVCs normally outweigh the costs. This conclusion, however, rests upon the relatively free movement of goods and services,^7^ and of people and capital worldwide. Some services may be delivered electronically (e.g. banking services), but most tangible goods need to be physically delivered from one location to another.^8^ This physical distribution—whether by road, rail, air and/or sea—involves people, and at least some of these people will need to cross national boundaries. Many GVCs also involve overseas subsidiaries of domestic multinational enterprises (MNEs), and these MNEs will require the international movement of staff and other employees as part of the regular management of their operations. Furthermore, several industrial sectors (e.g. food production) involve the substantial employment of seasonal migrant labour.

The Covid-19 pandemic has exposed the weaknesses in this model of doing business. First, expatriate staff and/or many of the people involved in the physical distribution of goods (truck drivers, seafarers, pilots etc.) may be directly affected by the virus, or may not be allowed to cross national borders. This will impede the effective operation of the GVCs. Second, international air travel has been severely circumscribed, even though most ports and sea routes remain open. Third, social distancing and other health checks create delays at borders. These disruptive effects become ever more severe, and the additional transaction costs larger, the greater the distances involved and the more borders that need to be crossed. These disruptive effects have highlighted sectors in which economies lack domestic productive capacity. Fourth, many firms (and governments) have experienced shortages of key goods and services, as foreign suppliers have favoured local customers. This discrimination has been particularly apparent in the supply of pharmaceuticals and medical equipment such as ventilators and personal protective equipment. Last but not least, the pandemic has exacerbated the long-standing scepticism of many people to free trade. Such sceptics include workers in many advanced economies who have seen their jobs offshored to the emerging economies in the recent past, as well as politicians and other people who abhor the pooling of sovereignty implied by the elimination of trade and investment barriers. Here it is illustrative to note the numerous comments by the US President (Donald Trump) about decoupling the US and Chinese economies and bringing manufacturing activities back to the United States, by the Australian PM^9^ (Scott Morrison) about nurturing local manufacture to ensure it is less reliant on global value chains, and by the Japanese PM^10^ (Shinzo Abe) about building an economy that is less dependent on China so that the nation can better avoid supply chain disruptions.

## The effects of the pandemic on firms’ GVC strategies

The Covid-19 pandemic will eventually be brought under control. People will go back to work. Consumption, and especially social consumption, will recover. Those firms that survive will restart production. Travel, both domestic and international, will resume. There will be numerous post-pandemic inquests seeking to establish the precise origins and epidemiological behaviour of the virus, and critically examining the responses of governments and supranational organizations (especially the World Health Organization) around the world in terms of containing the health effects of the virus and in mitigating the economic impacts of the counter-measures. It will become apparent that many governments were caught out by the speed and virulence with which Covid-19 took hold in their countries, and/or were also guilty of a lack of preparedness for such an eventuality. There will also be calls for governments to introduce better early warning mechanisms in their public health agencies, to restore indigenous manufacturing capabilities of essential products (e.g. vaccines and other pharmaceuticals), and to stockpile critical medical equipment and supplies.

But how should firms adjust their strategies, particularly with regard to the configuration of the GVCs in terms of the locations of the different activities and their governance—i.e. which activities are internalized (integrated) within the firm, and which are externalized? There will no doubt be much debate (both within firms and more widely) about how firms should build greater resilience through, on the one hand, reshoring GVC activities that had previously been offshored and, on the other hand, internalizing activities that had hitherto been undertaken by independent suppliers.

Reshoring may be achieved either by MNEs repatriating activities undertaken by foreign affiliates, or simply by firms replacing overseas suppliers of inputs with domestic suppliers. Reshoring should shorten supply chains, and make them less vulnerable to restrictions on the cross-border movement of people—though the supply chains will still be affected by any domestic restrictions on travel. Greater internalization of key activities might also bring potential benefits inter alia in terms of assured supply; improved scheduling and coordination; the elimination of opportunistic recontracting; and increased bargaining power vis-à-vis buyers and suppliers (Strange and Magnani 2018).

But would such significant reconfigurations of GVCs be merited? First, it is important to reiterate the cost and risk reduction benefits that firms derive from international diversification of their sources of supply. Reshoring may be a good idea if it allows firms to be closer and more responsive to the needs of their customers, but it heightens the exposure of firms to supply disruptions in their domestic economies. Furthermore, reshored activities may still require raw materials and other inputs that can only be sourced from overseas—hence reshoring may simply move reliance on imports further upstream in the GVC.^11^

The greater internalization of value-chain activities also has potential benefits, but these must be set against the additional costs. Greater externalization allows firms inter alia to focus on their core competences, and hence economize on their scarce financial and managerial resources; to have greater flexibility in response to volatile output demand; and to access cheaper and/or better quality inputs due to competition between outside suppliers (Strange and Magnani 2018). Certainly, firms should reevaluate the location and governance of their GVCs, but they need to weigh up the conflicting imperatives of robustness and efficiency. Firms might also consider alternative methods (e.g. establishing spare domestic capacity, stockpiling, greater liquidity, better risk management) of mitigating adverse effects on the functioning of their GVCs. But spare capacity, stockpiling and maintaining additional liquidity all involve opportunity costs.

Second, the economic impact of the Covid-19 pandemic has largely arisen from a combination of the supply shock and the demand shocks discussed above, and the severity of these shocks has varied by country according to the rates of infection and the counter-measures adopted by governments. Certainly, there have been serious supply chain shortages in some countries/sectors (notably, as noted above, in pharmaceuticals and medical equipment), but the impact such shortages needs to be evaluated alongside the impacts of the supply and demand shocks. It would make little sense to reconfigure GVCs if supply chain shortages were not the critical factor.

Third, the focus on supply chains draws attention away from the fact that many firms rely on foreign sales as well as domestic sales, and that diversification in revenue streams is also a means of reducing unsystematic risk. Reshoring of GVC activities may increase resilience in terms of domestic purchases, but it will concomitantly increase the costs of making foreign sales. FDI may be undertaken for efficiency-seeking reasons, but it may also be motivated by market-seeking, natural resource-seeking or strategic asset-seeking (Dunning and Lundan 2008). Furthermore, many firms try to manage their foreign exchange exposure by balancing their revenues and costs in different currencies, and reshoring selected activities might upset this balance.

Fourth, the Covid-19 pandemic is, as noted above, a global phenomenon, and both the health and the economic impacts have been experienced by most countries in the world. It would be feasible for firms to configure their GVCs in anticipation of epidemics that were likely to be localized (by diversifying into multiple countries) or diseases (e.g. malaria) that are endemic to certain countries (by either avoiding those countries, or taking appropriate preventative action). But the global spread of the Covid-19 virus took most people by surprise^12^ and, as noted above, different national governments reacted in different ways. Most firms will hopefully consider future pandemics as part of their routine risk assessment activities henceforth, but it is not certain that reshoring and internalization are the appropriate responses given that future pandemics are as likely to impact domestic economies as foreign locations. Rather, more international diversification and greater externalization are probably the answer. It has also been apparent from the Covid-19 pandemic that some countries (e.g. South Korea) were both more ready and responded more quickly and appropriately, than others. Firms might well be advised to consider such country-specific readiness and responsiveness as relevant location-specific advantages when making future location decisions.^13^

Fifth, and perhaps most importantly, the eventual reconfiguration of firms’ GVCs will to a large extent be determined by the responses of their national governments, and notably their attitudes towards conducting business with and in China. It is already apparent (see above) that the US, Australian and Japanese governments are looking explicitly to decouple their economies from their (inter)dependence on China, and this will no doubt be buttressed by a narrative looking to blame China for the onset of the pandemic. Furthermore, the Covid-19 pandemic has come at a time when globalization was already under threat because of concerns in many countries about sovereignty, national security and the unequal distribution of the benefits from globalization (Kobrin 2017; Aguilera et al. 2018; Rodrik 2018; Strange 2020).

## Final remarks

The Covid-19 pandemic has been global and indiscriminate. It has had a public health impact (if not equally) upon people in all countries (even indirectly in countries which have not reported confirmed cases) of all ethnicities, of all genders, and of every status in society, and given rise to restrictions on their freedom of movement. The pandemic has also had economic impacts as all countries have suffered supply and demand shocks to their national economies, and disruption to their international trade and investment flows. Furthermore, these adverse impacts—and national policies to mitigate their effects—have been felt beyond national borders.

Many firms, both large and small, have struggled to survive during the pandemic, and this will inevitably focus attention both on the apparent failings of their past business models and on how to build greater resilience in the future. The main conclusions from this paper are twofold. First, the widespread reshoring and/or internalization of GVC activities are unlikely to lead to greater resilience but may well necessitate substantial switching costs. Second, resilience will come from more, rather than less, diversification involving more suppliers in more countries, thus guarding against individual governments which close their borders to international movements of people, capital, goods and services. But—and this is the crucial consideration—the evolving geopolitical context and rising protectionist sentiments worldwide are likely to be the critical drivers.

There will also be new business opportunities. Many otherwise viable businesses will fail because of cash-flow problems, and these may well be acquisition targets for cash-rich firms and other investors. Krugman (2000) coined the term “fire-sale FDI” to refer to the flows of inward FDI into South Korea, Malaysia, Indonesia and other affected economies after the Asian Financial crisis of 1997–98. There are already reports that Gulf Sovereign Wealth Funds (SWFs) are mobilizing to buy shares in firms whose valuations have been hit hard by the Covid-19 pandemic.^14^ These possibilities have not escaped the attention of policy-makers. For instance, the European Commission warned Member States in March 2020^15^ to strengthen their vetting of foreign takeover bids, stressing that the Covid-19 pandemic had left “strategic assets” within the bloc vulnerable to acquisition from overseas. In India, the government tightened its foreign investment rules in April 2020 to block “opportunistic takeovers” of indigenous firms, especially by Chinese buyers.^16^

It would not be appropriate to end this paper without emphasizing once again the widespread human tragedy as a result of the Covid-19 pandemic. There are real dangers that many countries will adopt parochial and nationalist responses to the pandemic, and that poorer countries may suffer particularly with regard to food supply (World Food Programme 2020). But the Covid-19 pandemic is a global phenomenon, and its health impact cannot be confined to national borders. It will need concerted global cooperation to bring it under control. It is also to be hoped that national governments and relevant supranational organizations learn lessons from the Covid-19 pandemic, and take appropriate steps to reduce the likelihood of future pandemics, to improve the policy responses, and to minimize the adverse economic impacts.

## Footnotes

1 The UN Centers for Disease Control and Prevention (CDC) note that the term epidemic refers to an increase, often sudden, in the number of cases of a disease above what is normally expected in that population in that area, whilst pandemic refers to an epidemic that has spread over several countries or continents, usually affecting a large number of people. The distinction is important, both in terms of controlling the disease and in mitigating its economic impact.

2 BBC website, 18 April 2020.

3 More recent virus outbreaks such as the Severe Acute Respiratory Syndrome (SARS) epidemic in 2002–2004, the Middle East Respiratory Syndrome (MERS) epidemic in 2012, the Ebola epidemic in 2014–2016, and the Zika epidemic in 2015–2016 are all termed epidemics because their geographic reach was not global. Furthermore, the numbers of fatalities during these epidemics—though still high—are much lower than the fatalities already linked to the Covid-19 pandemic.

4 See the Oxford Covid-19 Government Response Tracker. Available at: https://www.bsg.ox.ac.uk/research/research-projects/coronavirus-government-response-tracker

5 Including the UK Prime Minister, Boris Johnson, who self-isolated between 25 March and 5 April 2020, was admitted to the Intensive Care Unit at St Thomas’ Hospital on 5 April, discharged from hospital on 12 April, and only returned to normal working on 27 April.

6 Buckley and Strange (2015) highlight how and why the international division of labour has progressed over the last 30 years.

7 Trade in many goods and services may be subject to tariffs and non-tariff barriers to trade, but firms will factor in such additional costs when establishing their GVCs.

8 Typical value chains involve the provision of many different goods and services in many different locations, and hence involve multiple deliveries across many national boundaries.

9 See Jamie Smyth, ‘Coronavirus shortages prompt Australia to bring manufacturing home.’ Financial Times, 15 April 2020. Available at: https://www.ft.com/content/04ac783d-8ced-4e66-9437-78b607cbd8d4.

10 See Walter Sim, ‘Coronavirus: Japan PM Shinzo Abe calls on firms to cut supply chain reliance on China.’ Straits Times, 16 April 2020. Available at: https://www.straitstimes.com/asia/east-asia/coronavirus-japan-pm-shinzo-abe-calls-on-firms-to-cut-supply-chain-reliance-on-china.

11 For instance, there are only a few countries in the world that are significant producers of the rare earths (e.g. neodymium, dysprosium, lanthanum, gadolinium, cerium) that are essential elements in many electronic products. Currently 85% of world production is in China, and 10% in Australia. The rare earths are essential because of their special electrochemical, magnetic and/or luminescent properties (Kirkpatrick 2019).

12 Not everyone was taken by surprise, and nor should they have been. Many commentators have long recognized the potential dangers of pandemics: see, for example, Bloom et al (2018), Fan et al (2018), Global Preparedness Monitoring Board (2019) and the March 2015 TED talk by Bill Gates. See the summary of the talk at the World Economic Forum website: https://www.weforum.org/agenda/2020/03/bill-gates-epidemic-pandemic-preparedness-ebola-covid-19/.

13 This will be particularly the case if the international role and funding of the World Health Organization (WHO) are eroded as a result of the Covid-19 pandemic.

14 Including Saudi Arabia’s Public Investment Fund, Abu Dhabi’s Mubadala and the Qatar Investment Authority. See Andrew England & Simeon Kerr, ‘Cash-rich Gulf funds hunt for bargains as asset prices plunge.’ (FT, April 16 2020). Available at: https://www.ft.com/content/3facc407-200f-4e7c-9914-79b4baece119?emailId=5e9dc563afa44b00049ab389&segmentId=f5ff1a1a-1590-1863-3861-e9c91a6a3b7f.

15 See the press release ‘Coronavirus: Commission issues guidelines to protect critical European assets and technology in current crisis’ from the European Commission, 25 March 2020. On 29 April 2020, the French economy minister announced an extended list of sectors deemed to be “strategic”, and reduced to a 10% stake the threshold for exercising a veto on non-European investors. Other EU countries implemented similar measures.

16 See Benjamin Parker, ‘India moves to curb Chinese corporate takeovers.’ Financial Times, 18 April 2020. Available at: https://www.ft.com/content/ad3f84b0-fb75-4588-97e8-4a657ad67883?emailId=5e9ed01df3399c0004daa49a&segmentId=2785c52b-1c00-edaa-29be-7452cf90b5a2.
